# Association of physical activity with bleeding events and safety in patients with haemophilia A starting emicizumab prophylaxis: an interim analysis of the TSUBASA study

**DOI:** 10.1007/s12185-023-03679-8

**Published:** 2023-12-15

**Authors:** Keiji Nogami, Teruhisa Fujii, Akihiro Sawada, Azusa Nagao, Chiai Nagae, Masanori Nojima, Nobuaki Suzuki, Daisuke Nosaka, Tomomi Shimura, Yoshimasa Sugao, Kagehiro Amano

**Affiliations:** 1 Department of Pediatrics, Nara Medical University, 840 Shijo-cho, Kashihara, Nara 634-8522 Japan; 2 Division of Transfusion Medicine/Hemophilia Treatment Center, Hiroshima University Hospital, Hiroshima, Japan; 3 Department of Respiratory Medicine and Hematology, Hyogo College of Medicine, Hyogo, Japan; 4 Department of Blood Coagulation, Ogikubo Hospital, Tokyo, Japan; 5 Department of Pediatrics, St. Marianna University School of Medicine, Kanagawa, Japan; 6 Center for Translational Research/Division of Advanced Medicine Promotion, Institute of Medical Science, University of Tokyo, Tokyo, Japan; 7 Department of Transfusion Medicine, Nagoya University Hospital, Aichi, Japan; 8 Chugai Pharmaceutical Co., Ltd, Tokyo, Japan; 9 Department of Laboratory Medicine, Tokyo Medical University, Tokyo, Japan

**Keywords:** Haemophilia A, Emicizumab, Patient-reported outcome, Physical activity

## Abstract

**Introduction:**

Little information exists on the relationship between bleeding outcomes and physical activity in patients with haemophilia A (PwHA).

**Aim:**

This interim analysis of the TSUBASA study (UMIN-CTR ID: UMIN000037448) evaluated the association of physical activity with bleeding and safety in PwHA starting emicizumab.

**Methods:**

PwHA without factor VIII inhibitors were recruited. Physical activity and bleed data were obtained using an electronic patient-reported outcome application and wearable activity tracker. Adverse events (AEs) were documented.

**Results:**

At data cut-off (31-May-2021), 107 PwHA were enrolled, with a median (range) age of 35 (0–73) years. Physical activity data were obtained for 74 participants. Of these, 47 (63.5%) recorded a total of 396 exercise events. The most common exercise events were walking (32.4%), cycling (14.9%), and football (5.4%). Two (0.5%) exercise events in the same individual were associated with bleeding (running, weight training).

The safety analysis population consisted of 106 participants treated with emicizumab (median observation period: 241.5 days). Twenty-one (19.8%) participants experienced a total of 39 AEs. Five (4.7%) experienced a serious AE, none of which was emicizumab-related, and three (2.8%) experienced an adverse drug reaction.

**Conclusions:**

PwHA receiving emicizumab in the TSUBASA study experienced minimal bleeding associated with physical activity.

**Trial registration:**

Trial registration: UMIN-CTR ID: UMIN000037448.

**Supplementary Information:**

The online version contains supplementary material available at 10.1007/s12185-023-03679-8.

## Introduction

When prophylactic treatment is inadequate, patients with haemophilia A (PwHA) develop chronic arthropathy due to repeated joint bleeding, which reduces their quality of life (QoL). In order to prevent the development of conditions such as chronic arthropathy, it is important for PwHA to achieve adequate haemostasis and maintain bone and joint health. Prior to the approval of factor (F)VIII replacement therapy, physical activity and participation in sports was discouraged in PwHA, due to the associated risk of bleeding [1]. However, with prophylactic treatment now readily available, participation in sports and exercise is encouraged, due to its associated health benefits such as decreased bleeding complications, improvement of cardiovascular function, and improved bone and joint health [2–7].

As a result of studies highlighting the benefits of exercise in PwHA, the World Federation of Haemophilia (WFH) guidelines encourage PwHA to participate in a variety of non-contact sports, including swimming, walking, jogging, golf, and table tennis [2]. Levels of physical activity in PwHA have been investigated in several studies, with reports that many PwHA have comparable fitness and activity levels to healthy individuals [8, 9]. Until recently, the gold standard treatment for haemophilia A (HA) was FVIII replacement therapy administered prophylactically [10]. Owing to this, the majority of studies have evaluated the risk of bleeding associated with exercise in PwHA receiving treatment with FVIII, with some consensus on the target trough activity level for periodic replacement therapy according to activity [1, 11–13].

The recombinant, humanised, bispecific monoclonal antibody emicizumab, is now available for the treatment of PwHA. Emicizumab substitutes for the function of activated FVIII by bridging activated FIX and FX, thereby improving haemostasis [14, 15]. In clinical trials, emicizumab prophylaxis demonstrated effective bleed control and was well tolerated in PwHA with or without FVIII inhibitors [16–19]. In addition, the subcutaneous administration of the drug has the advantage of extending the administration interval, and if physical activity can be maintained, it may contribute to an improvement in QoL compared with the more frequent replacement of conventional coagulation factor products. However, there are currently limited data evaluating the relationship between physical activity and bleeding outcomes in PwHA receiving emicizumab.

The TSUBASA study is designed to explore the relationship between physical activity and bleeding events, as well as safety and QoL, in PwHA initiating prophylactic treatment with emicizumab in a Japanese cohort.

## Materials and methods

### Study design and participants

TSUBASA is a prospective, multicentre, observational study conducted across 50 participating institutions in compliance with the International Conference on Harmonisation Guidelines for Good Clinical Practice, the Ethical Guidelines for Medical and Health Research Involving Human Subjects, and the Declaration of Helsinki. This ongoing study aims to enrol 160 participants with congenital HA without FVIII inhibitors to begin emicizumab prophylaxis, including at least 30 PwHA aged ≥ 6 years to < 18 years and at least 10 PwHA aged < 2 years. Eligible participants had diagnosis of congenital HA without active FVIII inhibitors at the time of enrolment, and were evaluated by the investigator, with emicizumab selected as the most appropriate treatment. Eligible participants must have also provided informed consent before entering the study. Individuals were excluded from participation in this study if they had an inherited or acquired bleeding disorder other than congenital HA, were undergoing immune tolerance induction treatment at the time of enrolment or have any other reason that, in the judgement of the investigator, would render the participant unsuitable for study participation. Participants who had previously been treated with emicizumab were also excluded to facilitate assessment of changes in QoL from before to after initiation of treatment and to also evaluate the safety of emicizumab in previously untreated PwHA.

Emicizumab was administered per the approved dosage regimens, which include a loading dose of 3 mg/kg every week (QW) for 4 weeks, followed by maintenance doses of 1.5 mg/kg QW, 3 mg/kg every 2 weeks, or 6 mg/kg every 4 weeks [20]. Treatment was continued until week 97 after the first dose, or until it becomes clinically inappropriate for the participant to continue treatment.

### Data collection

#### Physical activity

Exercise events were defined according to the Standards for Physical Activity for Health 2013 [21], in which all movement that uses more energy than used in a resting state is considered ‘physical activity’. To measure physical activity data, all participants ≥ 6 years of age continually wore an activity tracker while awake on the wrist of their non-dominant hand throughout five specified 8-day monitoring periods, which included the visit day (an on-site visit in which participants were monitored by the study administrator). Participants were asked to remove the activity tracker if they were going to be in water of ≥ 1 m in depth for more than 30 min. Data collection began from Week 5 after completing the loading dose period, and at 24-week intervals (weeks 25, 49, 73, and 97). The number of exercise events and duration of activity was recorded during these periods, in addition to metabolic equivalents (METs), which are the ratio of working metabolic rate relative to resting metabolic rate (one MET is equal to the energy expended when at rest). METs are used to classify exercise intensity (light exercise: < 3.0 METs; moderate: 3.0–6.0 METs; vigorous: > 6.0 METs) [22, 23]. Data management of activity monitoring was conducted using ActiGraph software.

#### Exercise status and bleeds attributable to exercise

Participants reported data on exercise status and bleeds related to exercise at week 5 and at 24-week intervals following enrolment (weeks 25, 49, 73, and 97) on an application installed on a mobile device [electronic patient-reported outcome (ePRO)] during the five specified 8-day monitoring periods in which the activity tracker was worn. Participants were permitted to have their responses entered by their caregiver if they were unable to enter the details themselves. These data entered by the participants or their caregivers were then compiled by the investigator.

#### Bleeds and medications used

All participants reported bleeds treated with a blood coagulation factor product, and the use of emicizumab and other haemophilia-related drugs, via the ePRO throughout the entire duration of the study. Bleeds related to surgical procedures were recorded in the ePRO or the electronic case report form (eCRF), which is completed by the investigator. Bleeding rate was measured as the number of bleeds requiring treatment with coagulation factor products over a given period of time.

#### Adverse events

Participants were asked to report information on adverse events (AEs) at each treatment visit, which was recorded by the investigator on the eCRF. The number of participants reporting AEs and the overall number of AEs was collected and evaluated according to system organ class, preferred term, and severity grade.

### Objectives and endpoints

The primary objective of the TSUBASA study was to explore the association between physical activity status and bleeding events in PwHA receiving emicizumab prophylaxis and to evaluate the quality and content of daily life during treatment with emicizumab. Additional objectives were to collect information on the safety of emicizumab and its efficacy in the treatment of previously untreated PwHA. For this interim analysis, only association between physical activity and bleeding, and safety, are reported.

The endpoints relating to physical activity and bleed outcomes are patient-reported exercise status and activity data from a wearable tracker and patient-reported status of bleeds attributable to exercise. Safety endpoints include the number of AEs, treatment-related AEs, and the development of FVIII inhibitors. The use of coagulation factors to prevent bleeding or maintain adequate haemostasis will be explored as an additional endpoint. Quality and content of daily life were also assessed; however, these endpoints are not reported in this interim analysis.

### Data analysis

The intent-to-treat population comprises all study participants enrolled per the ‘enrolment procedures’, other than those enrolled multiple times or in error. All participants who received ≥ 1 dose of emicizumab after enrolment were included in the safety analysis population, which was used to evaluate endpoints relating to bleed outcomes and the safety of emicizumab. The full analysis population at the time of this interim analysis comprises all eligible participants who have provided physical activity data; this population was used to evaluate endpoints related to patient-reported exercise status.

Participant clinical characteristics are reported using summary statistics; continuous variables are represented by means, standard deviations, medians, and ranges. Frequencies and proportions are used to describe categorical variables. For bleeding that required treatment with a blood coagulation factor, model-based annualised bleeding rates (ABRs) with confidence intervals (CIs) were generated using negative binomial regression.

## Results

### Participant demographics and clinical characteristics

**Fig. 1:**
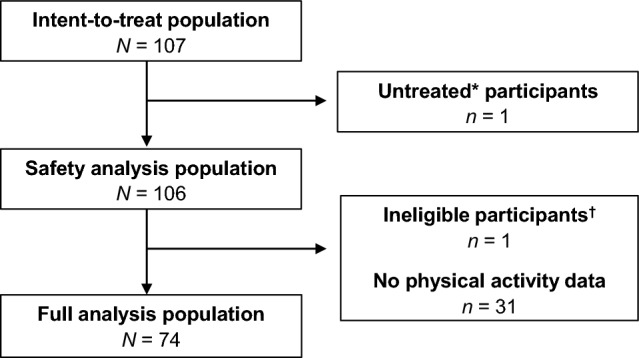
Participants included in the analyses. *Defined as no treatment with emicizumab. ^**†**^Participant was ineligible due to a dose interval deviation

At the time of the interim analysis data cut-off (31 May 2021), 107 participants had been enrolled since 1 November 2019. All participants were male, with a median age of 35 years (range 0–73 years). Of the 107 participants, 15 (14.0%) were < 2 years old (14 with severe HA, one with moderate HA). The safety analysis population comprised 106 participants (Fig. 1), with a median observation period of 241.5 days [interquartile range (IQR) 118–371 days]. Of these participants, bleeding in the 12 or 24 weeks before emicizumab administration was present in 13/15 (86.7%) participants < 2 years old, and 57/91 (62.6%) participants ≥ 2 years old. A diagnosis of severe HA was confirmed in 91 participants (85.8%), and 74 participants in the safety analysis population were previously treated with FVIII prophylaxis (69.8%); only six participants (5.7%) had not received previous treatment with any coagulation factor products (Table 1).

**Table 1:** Baseline characteristics of participants

Participant characteristics (intent-to-treat population)	*N* = 107
Male, *n* (%)	107 (100)
Age, median (range), years	35 (0–73)
HA severity*, *n* (%)	
Severe	91 (85.8)
Moderate	15 (14.2)
Bleeding history*, *n* (%)	
Aged < 2 years: bleeding present during the 12 weeks prior to study entry	13/15 (86.7)
Aged ≥ 2 years: bleeding present during the 24 weeks prior to study entry	57/91 (62.6)
Use of coagulation factor products prior to study*, *n* (%)	
None	6 (5.7)^†^
Prophylaxis	74 (69.8)
On-demand	26 (24.5)
Immune tolerance induction therapy*, *n* (%)	3 (2.8)
Target joints*, *n* (%)	19 (17.9)

Target joints were defined as joints with ≥3 bleeds during the 24 weeks prior to study entry. Target joints were not defined for participants <2 years old with no bleeding events in the 24 weeks prior to study entry

*Information missing for one participant (*N* = 106)

^†^Five of these participants were aged <2 years and the other had moderate HA. HA, haemophilia A

### Exercise status and bleeds

Of the 74 participants who comprised the full analysis population (Fig. 1), 47 performed 396 exercise events during the 8-day monitoring periods, as reported on the ePRO during the study visits. The median duration was 30.5 min (IQR 20–60) per exercise event. No exercise data were reported for 27 participants. Of the 396 events reported using the ePRO, 329 were collected by the wearable activity tracker (Table 2). For the 47 participants with exercise data, the most common physical activities performed during the study were walking (32.4%), cycling (14.9%), and football (5.4%) (Table 2). The exercise with the highest median average METs was basketball (5.72; range 5.27–6.06). A total of 18 bleeds were recorded across the 8-day monitoring periods. Only two (0.5%) of the 396 exercise events were associated with bleeding during the study, and both occurred in the same individual (Table 2). The events associated with a bleed were running (joint bleed in right knee) and weight training (intramuscular bleed in right upper arm), both occurring at week 49; each exercise event was 60 min in duration, with median (range) average METs of 2.55 (0.86–8.05) and 1.27 (0.86–2.60), respectively. The participant was a 29-year-old male with severe HA who had bleeding events present in the 24 weeks prior to initiation of emicizumab treatment and no target joints.

**Table 2:** Physical activity collected using the ePRO app and wearable activity tracker for participants ≥ 6 years of age (*N* = 74)

Exercise type	Number of PwHA, *n* (%)	Number of exercise events	Duration of exercise, median (range), minutes	Average METs, median (range)*	Maximum METs, median (range)*
Overall	47 (63.5)	329	30.0 (1–1240)	2.30 (0.86–8.05)	3.97 (0.86–14.19)
Walking	24 (32.4)	134	25.0 (1–783)	2.37 (0.99–7.96)	4.01 (1.25–11.26)
Movement not on motor list	23 (31.1)	58	30.0 (5–537)	1.67 (0.86–4.55)	3.11 (0.86–13.86)
Cycling	11 (14.9)	48	30.0 (5–1240)	2.39 (1.17–5.45)	3.73 (1.77–7.34)
Football	4 (5.4)	8	45.0 (15–150)	3.01 (0.86–5.60)	7.73 (0.86–9.71)
Golf	2 (2.7)	10	60.0 (8–315)	1.40 (0.86–2.71)	3.02 (0.86–6.07)
Running^†^	2 (2.7)	8	60.0 (9–60)	2.55 (0.86–8.05)	5.82 (0.86–10.19)
Skip rope	2 (2.7)	2	27.5 (10–45)	4.71 (3.76–5.66)	8.42 (5.79–11.05)
Basketball	2 (2.7)	7	60.0 (15–240)	5.59 (5.14–7.60)	10.68 (8.69–11.98)
Badminton	2 (2.7)	3	15.0 (15–25)	4.65 (2.78–4.69)	5.58 (4.17–9.20)
Bowling	2 (2.7)	2	90.0 (60–120)	2.60 (2.42–2.78)	4.76 (3.91–5.61)
Athletics	2 (2.7)	3	30.0 (30–35)	3.94 (3.56–4.16)	9.35 (7.45–11.65)
Fishing	1 (1.4)	11	175.0 (90–281)	1.41 (1.16–2.16)	3.09 (2.58–5.32)
Sambo	1 (1.4)	8	40.0 (15–45)	4.47 (2.14–5.36)	6.93 (2.64–7.56)
Tennis	1 (1.4)	7	60.0 (56–135)	3.86 (0.95–5.61)	8.36 (3.01–14.19)
Weightlifting	1 (1.4)	6	30.0 (9–60)	1.27 (0.86–2.60)	2.95 (0.89–4.21)
Darts	1 (1.4)	3	31.0 (25–35)	2.12 (1.73–2.48)	3.04 (2.82–3.37)
Baseball	1 (1.4)	3	127.0 (22–210)	5.72 (5.27–6.06)	10.82 (8.95–12.10)
Motorcycle sports	1 (1.4)	2	260.0 (220–300)	2.32 (2.28–2.36)	7.10 (5.60–8.61)
Radiological gymnastics	1 (1.4)	2	5.0 (5–5)	1.07 (0.86–1.28)	1.84 (0.86–2.82)
Weight training^†^	1 (1.4)	2	60.5 (60–61)	1.51 (1.50–1.52)	4.27 (3.56–4.98)
Climbing	1 (1.4)	1	30.0 (30–30)	3.76 (3.76–3.76)	5.63 (5.63–5.63)
Judo	1 (1.4)	1	10.0 (10–10)	3.94 (3.94–3.94)	6.33 (6.33–6.33)

^*^MET is the ratio of working metabolic rate relative to resting metabolic rate. One MET is equal to the energy expended when at rest

^†^Exercise events were associated with one bleeding event each in the same individual. MET, metabolic equivalent; PwHA, patients with haemophilia A

### Bleeding outcomes

Among the 106 participants in the safety analysis population, the overall median ABR was 0.91 (IQR 0.00–2.46); in participants < 2 years of age (*n* = 15), median ABR was 1.06 (IQR 0.00–1.46) (Table 3). The model-based ABR for the overall population was 2.3 (95% CI 1.6–3.3).

**Table 3:** Bleeding outcomes in the overall population and in participants < 2 years old receiving treatment with emicizumab

	Overall	Moderate HA	Severe HA
Overall population	*N* = 106	*n* = 15	*n* = 91
Annualised bleeding rate, median (IQR)	0.91 (0.00–2.46)	0.00 (0.00–1.23)	1.10 (0.00–2.55)
Model-based annualised bleeding rate*, mean (95% CI)	2.3 (1.6–3.3)	NA	NA
Participants with zero bleeds, n (%)	57 (53.8)	11 (73.3)	46 (50.5)
Participants < 2 years old	*N* = 15	*n* = 1	*n* = 14
Annualised bleeding rate, median (IQR)	1.06 (0.00–1.46)	1.46 (1.46–1.46)	1.04 (0.00–1.35)
Participants with zero bleeds, n (%)	9 (60.0)	0 (0.0)	9 (64.3)

^*^Model-based annualised bleeding rates are generated using negative binomial regression

*CI* confidence interval, *HA* haemophilia A, *IQR* interquartile range, *NA* not available

**Fig. 2:**
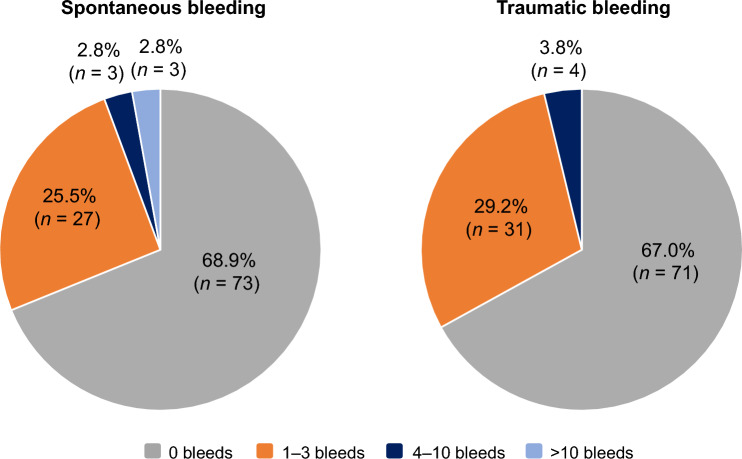
Number of participants with spontaneous or traumatic bleeding in the safety analysis population (*N* = 106)

**Fig. 3:**
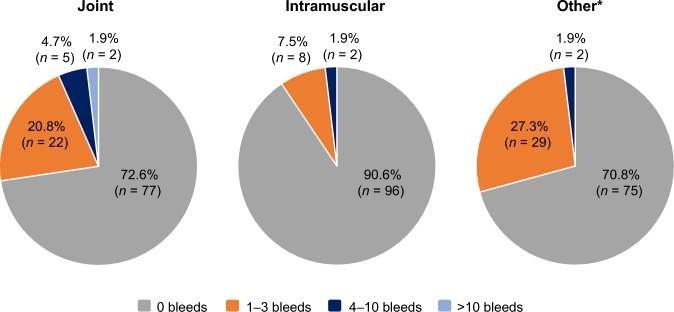
Number of participants with joint, muscle or ‘other’ bleeds in the safety analysis population (*N* = 106). *The locations of ‘other’ bleeds include the lower extremity, mouth, head, and face; a full list is provided in Supplementary Table

In total, 57/106 participants (53.8%) had zero bleeds during the observation period (median: 241.5 [IQR 118–371] days), including 11/15 participants (73.3%) with moderate HA and 46/91 participants (50.5%) with severe HA (Table 3). Overall, 31.1% of participants reported ≥ 1 incidence of spontaneous bleeding, and 33.0% reported ≥ 1 incidence of traumatic bleeding (Fig. 2). Joints and muscles were the most common bleed locations, with 27.4% and 9.4% of participants experiencing ≥ 1 joint or intramuscular bleed, respectively (Fig. 3).

### Safety

Overall, 39 AEs were reported in 21 (19.8%) of the 106 participants in the safety analysis population, including six who were < 2 years old (Table 4). Seven serious AEs were reported in five participants (4.7%); two of these individuals were < 2 years old. Serious AEs included: periodontitis, post-operative wound infection, anaemia, Kawasaki disease, sepsis, osteomyelitis, and necrotising fasciitis (*n* = 1 for each). No serious AEs were considered related to emicizumab treatment. Three participants (2.8%), all with severe HA and ≥ 2 years of age, experienced four adverse drug reactions. These were two incidences of injection-site erythema and two injection-site reactions. There were no deaths and no AEs leading to treatment discontinuation throughout the study duration.

**Table 4:** Safety summary in the overall population and in participants < 2 years old

	Overall	Moderate HA	Severe HA
Overall population	*N* = 106	*n* = 15	*n* = 91
Participants who experienced ≥ 1 AE, *n* (%)	21 (19.8)	2 (13.3)	19 (20.9)
Total number of AEs	39	2	37
Participants who experienced ≥ 1 SAE*, *n* (%)	5 (4.7)	1 (6.7)	4 (4.4)
Total number of SAEs	7	1	6
Participants who interrupted treatment due to SAEs, *n* (%)	1 (0.9)	0 (0.0)	1 (1.1)
Participants who discontinued treatment due to SAEs, *n* (%)	0 (0.0)	0 (0.0)	0 (0.0)
Participants who experienced ≥ 1 adverse drug reaction, *n* (%)	3 (2.8)	0 (0.0)	3 (3.3)
Total number of adverse drug reactions^†^	4	0	4
Participants who experienced ≥ 1 serious adverse drug reaction, *n* (%)	0 (0.0)	0 (0.0)	0 (0.0)
Participants who interrupted or discontinued treatment due to adverse drug reactions, *n* (%)	0 (0.0)	0 (0.0)	0 (0.0)
Participants < 2 years old	*N* = 15	*n* = 1	*n* = 14
Participants who experienced ≥ 1 AE, *n* (%)	6 (40.0)	1 (100.0)	5 (35.7)
Total number of AEs	10	1	9
Participants who experienced ≥ 1 SAE* n* (%)	2 (13.3)	0 (0.0)	2 (14.3)
Total number of SAEs^‡^	2	0	2
Participants who interrupted or discontinued treatment due to SAEs, *n* (%)	0 (0.0)	0 (0.0)	0 (0.0)
Participants who experienced ≥ 1 adverse drug reaction, *n* (%)	0 (0.0)	0 (0.0)	0 (0.0)
Total number of adverse drug reactions	0	0	0
Participants who experienced ≥ 1 serious adverse drug reaction, *n* (%)	0 (0.0)	0 (0.0)	0 (0.0)
Participants who interrupted or discontinued treatment due to adverse drug reactions, *n* (%)	0 (0.0)	0 (0.0)	0 (0.0)

*AE* adverse event, *HA* haemophilia A, *SAE* serious adverse event

*SAEs included periodontitis, post-operative wound infection, anaemia, Kawasaki disease, sepsis, osteomyelitis, necrotising fasciitis

^†^Adverse drug reactions included an injection-site reaction and an injection-site erythema in one participant, an injection-site reaction in another participant, and an injection-site erythema in a third

^**‡**^The two SAEs in participants < 2 years old were anaemia and Kawasaki disease

### Use of other haemostatic agents

In the safety analysis population (*N* = 106), 74 participants (69.8%) received a combined total of 584 doses of non-emicizumab haemostatic agents during the study (Table 5). Of these doses, 284 (48.6%) were administered to maintain adequate haemostatic potential. The most commonly used FVIII agent was rurioctocog alfa pegol, with 23 participants (21.7%) receiving ≥ 1 dose during the study.

**Table 5:** Use of other haemostatic agents throughout the study

Name of coagulation factor	Participants receiving ≥ 1 dose, *n* (%)	Total doses given, *n*	Purpose of use		Dose (IU/kg), median (range)
			Haemostasis, *n* (%)	Other, *n* (%)	
Overall	74 (69.8)	584	284 (48.6)	300 (51.4)	NA
Rurioctocog alfa pegol	23 (21.7)	169	126 (74.6)	43 (25.4)	28.78 (19.4–80.0)
Rurioctocog alfa	18 (17.0)	86	27 (31.4)	59 (68.6)	37.88 (15.0–146.2)
Efraloctocog alfa	17 (16.0)	163	40 (24.5)	123 (75.5)	51.90 (26.6–68.3)
Octocog beta	8 (7.5)	31	7 (22.6)	24 (77.4)	25.03 (20.7–62.1)
Lonoctocog alfa	5 (4.7)	9	4 (44.4)	5 (55.6)	34.40 (19.3–52.1)
Turoctocog alfa	5 (4.7)	83	47 (56.6)	36 (43.4)	28.90 (20.2–120.5)
Cross eight	2 (1.9)	8	3 (37.5)	5 (62.5)	17.24 (16.6–33.2)
Octocog alfa	2 (1.9)	4	3 (75.0)	1 (25.0)	30.76 (29.0–31.3)
Tranexamic acid	1 (0.9)	27	27 (100.0)	0 (0.0)	NA
Damoctocog alfa pegol	1 (0.9)	4	0 (0.0)	4 (100.0)	38.36 (38.4–38.4)

*IU* international unit, *NA* not available

## Discussion

Overall, PwHA took part in a wide variety of physical activities, and the majority of participants in the TSUBASA study (46/47 participants; 97.9%) did not experience bleeds associated with physical activity at the time of this interim analysis.

Only one participant reported bleeds related to exercise during the study with both exercise events having a median average METs score of < 3.0. The individual who reported bleeds also experienced bleeding events in the 24 weeks prior to emicizumab initiation. It is unknown whether the prior bleeding events were associated with exercise. The results of this interim analysis suggest that emicizumab provides adequate coverage in PwHA to prevent bleeding events during physical activity.

Previous studies have assessed the level of physical activity among PwHA treated with FVIII prophylaxis, evaluating the association of exercise with bleeding outcomes. In a study conducted by Pierstorff et al*.* in children and adolescents treated with FVIII prophylaxis, no participant experienced a joint or muscle bleed as a result of an exercise event [24]. Similarly, in a retrospective study conducted in adolescents receiving FVIII prophylaxis by Ross et al. [11] level of athletic participation was not a significant prognostic factor for joint haemorrhage when analysed using a logistic regression model. It was noted that the frequency of joint haemorrhages and new injuries did not significantly differ between PwHA who conducted high-impact athletics (e.g. basketball, baseball, tennis) and those who participated in low-impact athletics (e.g. golf, walking, cycling) [11].

Overall, in the TSUBASA study, the median ABR was low (0.91), with 54% of participants experiencing zero bleeds during the study period. These results contribute to the library of evidence supporting the efficacy of emicizumab in the clinical trial setting [16–19]. Additionally, no new safety signals were identified for emicizumab prophylaxis during this evaluation period, and no discontinuation of treatment was recorded.

The results of this interim analysis in PwHA receiving emicizumab align with results from previous studies in PwHA receiving FVIII prophylaxis, indicating that exercise and physical activity do not appear to increase the incidence of bleeding, regardless of the type of prophylaxis received [1, 11, 13]. Data generated from this study regarding QoL and the development of FVIII inhibitors will be reported in the final analysis.

### Limitations of the study

This study has several limitations, with the first being that the reasoning for administration of FVIII coagulation products, other than to maintain haemostasis, was not reported. Additionally, this study is an exploratory study and not a randomised controlled trial; therefore, low bleeding rates cannot be conclusively attributed to treatment with emicizumab prophylaxis, due to the lack of a comparator arm (e.g. FVIII prophylaxis or no prophylaxis) and the potential use of concurrent FVIII prophylaxis prior to participation in exercise. Assessment of physical activity and bleeding events was based on patient-reported outcomes, which are subject to participant inertia and interpretation.

Finally, bleeding events during exercise were only recorded during five defined 8-day periods; bleeds attributable to exercise outside of these windows were not monitored. This was done to ensure compliance in wearing the device as, at the time when the study began, the use of wearable devices was not widespread in Japan. Hence, representativeness of the real physical activity is limited, and this may lead to the performance of physical activity only during those days. Therefore, the rate of activity-related bleeds may be underestimated.

## Conclusions

The results of this study provide further evidence of the effectiveness and safety of emicizumab. A variety of exercise types were recorded throughout this study, with only two events being associated with one bleeding occurrence each. These results suggest that the health benefits of physical activity for PwHA receiving emicizumab may outweigh the associated bleeding risk, which appears to be minimal.

### Supplementary Information

Below is the link to the electronic supplementary material.

Supplementary file1 (DOCX 13 kb)

## Data Availability

Data are available upon reasonable request.
