# Supplementary material for: Association of physical activity with bleeding events and safety in patients with haemophilia A starting emicizumab prophylaxis: an interim analysis of the TSUBASA study
Source: Int J Hematol. 2023 Dec 15;119(1):14–23. doi: 10.1007/s12185-023-03679-8 (PMC10769904; doi:10.1007/s12185-023-03679-8)
Supplement: Supplementary file 1 — Supplementary file1 (DOCX 13 kb) [file 12185_2023_3679_MOESM1_ESM.docx]

**Supplementary Material**

**Supplementary Table 1.** Numbers of bleeds that occurred in locations other than joints or muscles

|  | **Number of bleeds** | | |
| --- | --- | --- | --- |
| **Bleed location** | **Overall**  **(*N* = 106)** | **Moderate HA**  **(*n* = 15)** | **Severe HA**  **(*n* = 91)** |
| Lower extremity | 13 | 1 | 12 |
| Mouth | 9 | 0 | 9 |
| Head | 7 | 0 | 7 |
| Face | 5 | 2 | 3 |
| Hand | 4 | 0 | 4 |
| Back | 4 | 0 | 4 |
| Stool | 4 | 0 | 4 |
| Hip | 2 | 0 | 2 |
| Nasal | 2 | 0 | 2 |
| Neck | 1 | 0 | 1 |
| Arm | 1 | 0 | 1 |
| Other | 1 | 0 | 1 |
